# Unvaccinated Children Are an Important Link in the Transmission of SARS-CoV-2 Delta Variant (B1.617.2): Comparative Clinical Evidence From a Recent Community Surge

**DOI:** 10.3389/fcimb.2022.814782

**Published:** 2022-03-08

**Authors:** Hongru Li, Haibin Lin, Xiaoping Chen, Hang Li, Hong Li, Sheng Lin, Liping Huang, Gongping Chen, Guilin Zheng, Shibiao Wang, Xiaowei Hu, Handong Huang, Haijian Tu, Xiaoqin Li, Yuejiao Ji, Wen Zhong, Qing Li, Jiabin Fang, Qunying Lin, Rongguo Yu, Baosong Xie

**Affiliations:** ^1^ Department of Respiratory and Critical Care Medicine, Fujian Provincial Hospital, Fujian Shengli Medical College, Fujian Medical University, Fuzhou, China; ^2^ Department of Orthopaedics, Affiliated Hospital of Fujian Putian University, Putian, China; ^3^ College of Mathematics and Statistics, Fujian Normal University, Fuzhou, China; ^4^ Nursing Department, Fujian Provincial Hospital, Fuzhou, China; ^5^ Department of Respiratory and Critical Care Medicine, The First Affiliated Hospital of Fujian Medical University, Fuzhou, China; ^6^ Department of Respiratory and Critical Care Medicine, Affiliated Hospital of Putian University, Putian, China; ^7^ Department of Pediatrics, Fujian Maternal and Child Health Hospital, Fuzhou, China; ^8^ Fujian Hospital of Shanghai Children’s Medical Center, Fuzhou, China; ^9^ Department of Internal Critical Medicine, Affiliated Hospital of Putian University, Putian, China; ^10^ Department of Laboratory Medicine, Affiliated Hospital of Putian University, Putian, China; ^11^ Department of Surgical Critical Medicine, Fujian Provincial Hospital, Fuzhou, China

**Keywords:** COVID 19, children, delta variants, clinical features, vaccination

## Abstract

**Objective:**

To evaluate the necessity of Covid-19 vaccination in children aged < 12 y by comparing the clinical characteristics between unvaccinated children aged < 12 y and vaccinated patients aged ≥ 12y during the Delta surge (B.1.617.2) in Putian, Fujian, China.

**Methods:**

A total of 226 patients with SARS-Cov-2 Delta variant (B.1.167.2; confirmed by Real-time PCR positivity and sequencing) were enrolled from Sep 10th to Oct 20th, 2021, including 77 unvaccinated children (aged < 12y) and 149 people aged ≥ 12y, mostly vaccinated. The transmission route was explored and the clinical data of two groups were compared; The effect factors for the time of the nucleic acid negativization (NAN) were examined by R statistical analysis.

**Results:**

The Delta surge in Putian spread from children in schools to factories, mostly through family contact. Compared with those aged ≥ 12y, patients aged < 12y accounted for 34.07% of the total and showed milder fever, less cough and fatigue; they reported higher peripheral blood lymphocyte counts [1.84 (1.32, 2.71)×10^9/L vs. 1.31 (0.94, 1.85)×10^9/L; *p*<0.05), higher normal CRP rate (92.21% vs. 57.72%), lower IL-6 levels [5.28 (3.31, 8.13) vs. 9.10 (4.37, 15.14); *p*<0.05]. Upon admission, their COVID19 antibodies (IgM and IgG) and IgG in convalescence were lower [0.13 (0.00, 0.09) vs. 0.12 (0.03, 0.41), *p*<0.05; 0.02 (0.00, 0.14) vs. 1.94 (0.54, 6.40), *p*<0.05; 5.46 (2.41, 9.26) vs. 73.63 (54.63, 86.55), *p*<0.05, respectively], but longer NAN time (18 days vs. 16 days, *p*=0.13).

**Conclusion:**

Unvaccinated children may be an important link in the transmission of SARS-CoV-2 delta variant (B1.617.2), which indicated an urgent need of vaccination for this particular population.

## Introduction

Since its outbreak in early 2020, Covid-19 has brought about a global pandemic, with a cumulative total of over 235,634,315 infections and a toll of tens of thousands of deaths (4,973,007 in total as of October 27, 2021). Amid the development of the Covid-19 pandemic, several mutants of the virus have been discovered, including Alpha (the B.1.1.7 in the United Kingdom), Beta (the B.1.351 in South Africa), Gamma, Lamda, Delta etc., among which the Delta variant (B.1.617.2) was first identified in Maharashtra in late 2020 (Cherian et al., 2021) and spread to at least more than 180 countries or regions, making it the most aggressive and prevalent variant around the world. The first locally-transmitted case of Delta variant in China was reported in Guangzhou on May 21, 2021, which was introduced by imported overseas individuals and resulted in a total of 167 localized cases. So far, four more surges have been reported successively in Yunnan Ruili Airport, Nanjing Lukou Airport, Zhengzhou and Putian. Studies have shown that compared with the wild strain, the Delta variant showed characteristics of a shorter incubation period (4.7 vs. 6.3 days), faster transmission, higher viral load (10^6 vs. 10^4 copies) and significantly longer time for nucleic acid negativization (NAN), and that the condition exacerbated 2.98 times faster and was 1.45 times more likely to deteriorate into a critical status in the elderly patient population (Li et al., 2021). The global launch of the vaccination against COVID-19 has been effective for the Delta variant, with Pfizer bioNTech reducing the symptomatic population from 94% to 64% and Oxford Astrazea from 73% to 60% (Baraniuk, 2021) and Moderna vaccine (mRNA 1273) preventing 81% of hospitalizations and 76% of infections and Pfizer bioNTech (BNT162B2) vaccine reducing 75% of hospitalizations and 42% of infections (Grannis et al., 2021). Several studies have also shown that vaccination provided 90% protection for frontline workers (Fowlkes et al., 2021). The CDC in the United States cautioned that people who had not been vaccinated would face an infection risk that was 11 times higher (Dyer, 2021b). All these findings suggested that the Covid-19 nucleic acid vaccination can provide effective protection from Delta variant infection and prevent it from developing into severe diseases. On the other hand, the L452R and P681R mutants of Delta (B.1.617.2) enhance its binding to the ACE receptor on human alveolar epithelial cells, inducing faster transmission, and trigger immune escape, resulting in compromised vaccination (Chia et al., 2021; Davies et al., 2021; Dyer, 2021a; Pritchard et al., 2021; Pung et al., 2021). Studies showed that after vaccination, the viral load in patients infected with the Delta variant was equivalent to that of unvaccinated patients (Griffin, 2021). Moreover, the protection against Delta variant was weaker than that against other mutant strains, which may result in breakthrough cases (Mlcochova et al., 2021), posing new challenges to the global efforts in containing the Covid-19 pandemic.

At present, vaccination programs for adolescents (≥12 years of age) have been approved and implemented in countries such as China, the United States, and the United Kingdom, but no mass vaccination has been conducted globally for children and adolescents under the age of 12 years due to remaining controversies over the risk-benefit issues of vaccination in children and adolescents (“Coronavirus (COVID-19) Update: FDA Authorizes Pfifizer-BioNTech COVID-19 Vaccine for Emergency Use in Adolescents in Another Important Action in Fight Against Pandemic 2021.,” 2021; “Press Release: JCVI Issues Advice on COVID-19 Vaccination of Children and Young People.,” 2021). Studies showed that children generally had favorable prognosis and only experience mild symptoms, ranging from symptoms of acute upper respiratory tract infection, mild fever, to digestive symptoms such as nausea, vomiting, abdominal pain, and diarrhea, which may account for the hesitation of a vaccination need for this particular population (Schroeder et al., 2020). On the other hand, some scholars believed that the negligence of these infected children often turned them into hidden spreaders of the virus and aggravated the epidemic (Yonker et al., 2020). A study once proposed a mathematical model of Susceptible-Exposed-Infectious-Recovered (SEIR) disease transmission with the United Kingdom as an example, which projected that if adolescents and children are included in the vaccination program, the overall COVID-19 mortality rate will be reduced by 57% and the number of long-term COVID-19 infection cases will be reduced by 75% (Shiri et al., 2021). The findings from this study suggested that vaccination in children may play an important role in reducing the overall morbidity and mortality of COVID-19. According to the current vaccination policy in China, the children under 12 years of age in this Putian surge (accounting for over 30% of the total) were not yet vaccinated against Covid-19 while most patients over 12 years of age were vaccinated, so a comparative analysis of the epidemic and its characteristics in this population would help evaluate the necessity of vaccination in children under 12 years of age.

## Materials And Methods

### Study Design and Participants

The study enrolled a total of 226 individuals with positive SARS-CoV-2 Delta (B.1.617.2) (confirmed by SARS-CoV-2 PCR), who were admitted to the designated hospital (The Affiliated Hospital of Putian University) from September 10 to October 20, 2021. Relevant information was collected regarding the patients’ epidemiological details, clinical data, laboratory results, kinetics of viral load, and information of last negative nucleic acid test. According to the current vaccination policy of China, the individuals were divided into two groups: Group 1 (aged<12y), who had not been vaccinated, and Group 2 (aged ≥12y), most of whom had been vaccinated with Chinese inactivated vaccine from Sinopharm or Sinovac company.

### Data Collection

Standardized data collection forms were employed to collect, from electronic medical records, the clinical and laboratory data, including clinical manifestations, laboratory results, treatment and prognosis. In terms of laboratory data, the cycle threshold (CT) value was measured by SARS-Cov-2 real-time PCR and the serological results included blood test, biochemical analysis, CRP, IL-6, SARS-CoV-2 IgG antibody titer, blood coagulation function, and microbe testing. The above serological tests were performed 24 hours after admission and antibody in convalescence was tested within 10 to 20 days after admission. The first day of onset was designated as the day of COVID-19 diagnosis for asymptomatic patients and the day of COVID-19 symptoms or the day of COVID-19 diagnosis (whichever was earlier) for symptomatic patients. From the 10th day of the disease course, nasopharyngeal swab testing was performed every day until a negative nucleic acid testing was successively reported twice with an interval of over 24 hours. The time for NAN was defined as the duration from the first day of positive nucleic acid report to the time of the second negative nasopharyngeal swab testing of the two successive negative nucleic acid tests (“Diagnosis and treatment protocol for novel coronavirus pneumonia (Trial Version 8).”, 2020).

### Viral RNA Sequencing

Fujian Provincial CDC performed the second-generation whole genome sequencing of the SARS-Cov-2 specimens by positive fluorescence real-time PCR. The epidemic strain was confirmed to be the Delta variant (B.1.617.2), with which all patients were infected.

### Clinical Management

All enrolled COVID-19 patients were admitted to the hospital, quarantined, and monitored for their vital signs, body temperature and respiration. Patients whose SpO_2_ was lower than 93% were treated with oxygen therapy. Most patients were treated with Chinese traditional medicine. Patients with fever or severe illness received the treatment of BRII 196/198, a neutralizing antibody, after informed consent was obtained. According to the national guidelines, glucocorticoids were only suitable for specific people, and antibiotics were used for patients with bacterial infections. In terms of disease severity, all patients were classified as asymptomatic, mild (no pneumonia by chest radiography), moderate (pneumonia by chest CT), severe (SpO_2_<93%; oxygen therapy required), or critical (RICU admission or mechanical ventilation required). Patients were discharged when the discharging standards were met (“Diagnosis and treatment protocol for novel coronavirus pneumonia (Trial Version 8).”, 2020). The collection of clinical data was reviewed at discharge.

### Statistical Analysis

All data were analyzed with the SPSS 23 software. The data of continuous parameters were expressed as mean (variance), or median (interquartile range, IQR), and assessed by T-test for normal variables or by Wilcoxon rank sum test for non-normal variables. Categorical variables were presented as frequency (percentage) and analyzed by Chi Square test and Fisher’s precision probability test. The NAN-related factors were estimated by the least-absolute method with R data analysis software. Two-tailed *p* value of less than 0.05 was considered statistically significant.

### Ethics Approval

All patients signed informed consent and the ethics approval was granted by the Ethics Committee of the Affiliated Hospital of Putian University (No.202152).

## Results

### Baseline Information of the Patients

A total of 226 infected patients were enrolled, of which 77 cases were under the age of 12 years (34.07%), with 44 males (57.14%) and a median age of nine years [6, 9], and 149 cases were aged ≥12 years (65.93%), with 50 males (33.56%) and a median age of 39 years [32, 50]. The children aged <12y were unvaccinated and had no underlying conditions, while 94.63% (141/149) of patients aged ≥12 years were vaccinated, of whom nine (6.04%) received one dose of vaccine and 132 (88.59%) received two doses of vaccine and 18 (12.08%) reported coexisting conditions (11 with diabetes, six hypertension, and one chronic kidney disease) (**Table 1**). On admission, the detected RT-PCR cycle threshold values (CT value) of children aged <12y were almost the same as that of patients aged ≥12y [ORF1 lab value: 21.28 (17.74, 26.15) vs. 23.21 (17.09, 27.51), *p*=0.38; N value: 20.68 (15.93, 25.33) vs. 22.50 (15.59, 26.79), *p*=0.46] (**Table 2**).

**Table 1:** Baseline information of 226 individuals infected with SARS-CoV-2 Delta B.1.167.2.

	All patients(n = 226)	<12(n=77)	>=12(n=149)
Age	32 [9,46]	9 [6,9]	39 [32,50]
Gender			
Male	94	44 (57.14%)	50 (33.56%)
Female	132	33 (42.86%)	99 (66.44%)
Vaccinated(%)	141	0 (0.00%)	141 (94.63%)
One-dose vaccination	9	0 (0.00%)	9 (6.04%)
Two-dose vaccination	132	0 (0.00%)	132 (88.59%)
Basic illness	18	0 (0.00%)	18 (12.08%)
Diabetes	11	0 (0.00%)	11 (7.38%)
Hypertension	6	0 (0.00%)	6 (4.03%)
Cardiovascular diseases	0	0 (0.00%)	0 (0.00%)
Chronic liver diseases	0	0 (0.00%)	0 (0.00%)
Respiratory diseases	0	0 (0.00%)	0 (0.00%)
Nervous system diseases	0	0 (0.00%)	0 (0.00%)
Blood system diseases	0	0 (0.00%)	0 (0.00%)
Chronic kidney diseases	1	0 (0.00%)	1 (0.67%)
Metabolic disease	0	0 (0.00%)	0 (0.00%)
Tumor	0	0 (0.00%)	0 (0.00%)

**Table 2:** Comparative analysis of clinical features of SARS-Cov-2 Delta (B.1.167.2) infection between children aged<12y and patients aged≥ 12y.

	All patients (n = 226)	<12 (n = 77)	≥12 (n = 149)	P-value
**Clinical features**				
Fever n(%)	147	58 (75.32%)	89 (59.73%)	0.02
Degrees of Fever n(%)				0.05
Low fever n(%)	61	31 (40.26%)	30 (20.13%)	
Moderate fever n(%)	58	21 (27.27%)	37 (24.83%)	
High fever n(%)	27	7 (9.09%)	20 (13.42%)	
Cough n(%)	80	14 (18.18%)	66 (44.30%)	<0.001
Expectoration n(%)	42	6 (7.79%)	36 (24.16%)	0.002
Nasal congestion n(%)	3	0 (0.00%)	3 (2.01%)	0.553
Sore throat n(%)	4	0 (0.00%)	4 (2.68%)	0.302
Dyspnea n(%)	3	0 (0.00%)	3 (2.01%)	0.553
Fatigue n(%)	64	13 (16.88%)	51 (34.23%)	0.006
Inappetence n(%)	8	2 (2.60%)	6 (4.03%)	0.719
Headache or Sore muscle n(%)	13	3 (3.90%)	10 (6.71%)	0.55
Diarrhea n(%)	7	2 (2.60%)	5 (3.36%)	1
Fever onset after diagnosis Median(IQR)	1 [1,4]	1[1,3]	2 [1,4]	0.31
Fever time Median (IQR)	1 [0,3]	1 [0,2.5]	1 [0,3.5]	0.998
CT value at diagnosis (ORF1lab)	22.20 [17.27, 27.26]	21.28 [17.74, 26.15]	23.21 [17.09, 27.51]	0.38
CT value at diagnosis (N)	21.90 [15.61, 26.32]	20.68 [15.93, 25.33]	22.50 [15.59, 26.79]	0.463
**Disease severity**				0.127
Asymptomatic	5	5 (6.49%)	0 (0.00%)	——
Mild	83	34 (44.16%)	49 (32.89%)	——
Moderate	132	38 (49.35%)	94 (63.09%)	——
Severe	4	0 (0.00%)	4 (2.68%)	——
Critical	2	0 (0.00%)	2 (1.34%)	——
Laboratory diagnostics				
WBC count(x10^9/L)average (SD)	6.25 (2.01)	5.89 (1.90)	6.45 (2.04)	0.05
Lymphocyte count(x10^9/L)Median (IQR)	1.46 [1.02,2.22]	1.84 [1.32,2.71]	1.31 [0.94,1.85]	<0.001
CRP<5mg/l	157	71 (92.21%)	86 (57.72%)	<0.001
IL-6(pg/ml)	7.00 [3.82, 13.98]	5.28 [3.31,8.13]	9.10 [4.37,15.14]	<0.001
**Treatment and prognosis**				
Pneumonia n(%)	135	41 (53.25%)	94 (63.09%)	0.12
Respiratory failure n(%)	6	0 (0.00%)	6 (4.03%)	0.302
Intubation rate, n(%)	2	0 (0.00%)	2 (1.34%)	0.549
TCM treatment, n(%)	214	67 (87.01%)	147 (98.66%)	<0.001
Antibiotic usage rate, n(%)	15	7 (9.09%)	8 (5.37%)	0.398
Neutralizing antibody of BRII, n(%)	42	0 (0.00%)	42 (28.19%)	<0.001
Convalescent plasma therapy, n(%)	5	0 (0.00%)	5 (3.36%)	0.169
Hormone therapy, n(%)	2	0 (0.00%)	2 (1.34%)	0.549
ICU occupancy	5	0 (0.00%)	5 (3.36%)	0.315
Nucleic acid negativization time(days),Median (IQR)	16[12,22]	18 [13,23.5]	16 [12,21]	0.13
Death rate, n(%)	0	0 (0.00%)	0 (0.00%)	1

1. Data include average (SD), median (IQR), n (%) or n/N (%). N is the number of patients with available data. 2. The category variable is “number of people (proportion)”. In numerical variables, the normal variable is “mean (standard deviation)” and the non-normal variable is “median (quartile spacing, quartile spacing)”.

### Transmission Route of SARS-CoV-2 Delta (B.1.617.2) in Putian

**Figure 1:**
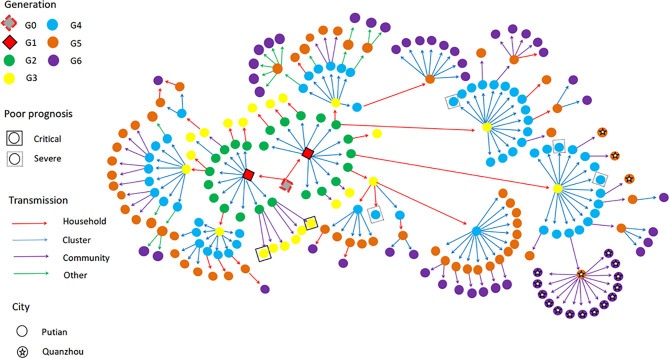
The transmission network of SARS-Cov-2 Delta (B.1.167.2) in the Putian surge. The transmission chain of the 226 infected patients was shown in the network. Each transmission generation is shown in rhombus or circles with different colors. The first-generation patients (rhombus with black solid line, G1) were in the middle. G1 was phylogenetically linked to an imported case (rhombus with red dotted line, G0). Colored arrows indicate different transmission routes. The transmission includes household, cluster (at school and factory), community (sporadic case in the community) and others (work and social contacts). Severe (dotted line) and critical (solid line) patients were labelled with squared shapes. Asterisks indicate patients in or to other cities (For interpretation of the references to color in this figure legend, please refer to the web version of this article).

In this local surge in Putian, Fujian, the first index case was a middle-aged male, who was infected during quarantine after entry into China and transmitted the SARS-CoV-2 Delta (B.1.617.2) to his two children (G1). The activities of the two children in the school spread the virus to their classmates (G2), who extended the transmission to their family members (G3). The latter transmitted the virus to the factories (G4) where they worked. All of these cases were epidemiologically or genetically traced back to the first case (The specific transmission route is shown in **Figure 1**).

### Comparison of Clinical Characteristics Between Patients Aged <12y and Those Aged ≥12 y

Compared with patients aged **≥**12y, 75.32% (58/77) of children aged <12y developed milder fever (*p*<0.05), minor symptoms such as cough (18.18% vs. 44.30%, *p*<0.05), expectoration (7.79% vs. 24.16%, *p*<0.05) and fatigue (16.88% vs. 34.23%, *p*<0.05). But no significant difference was found in the day of fever onset after diagnosis [1 (1, 3) vs. 2 (1, 4)] and duration of fever [1 day (0, 2.5) vs. 1 day (0, 3.5), *p*>0.05] (**Table 2**).

**Figure 2:**
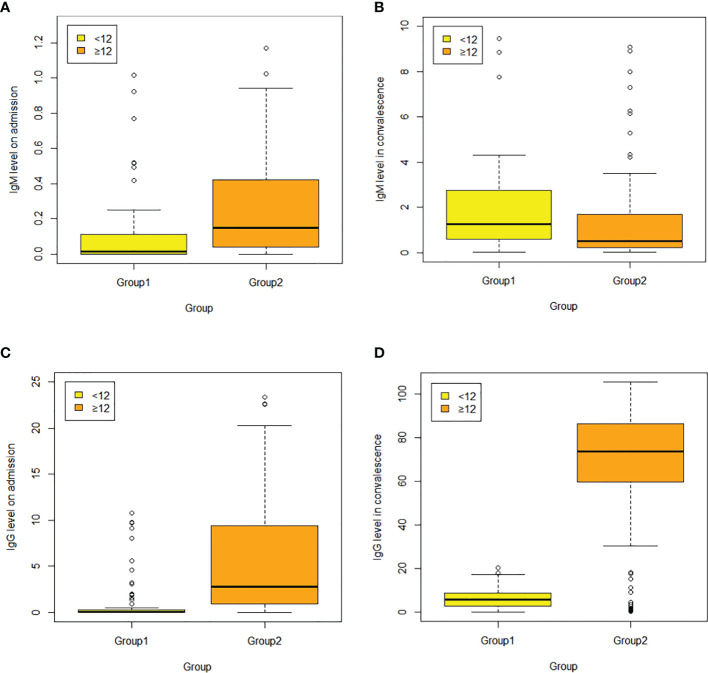
**(A–D)** Serum SARS-CoV-2 IgM and IgG levels between the two groups were compared on admission and in convalescence respectively. Dots represent IgM/IgG level in children aged <12 years (group1) and those aged≥ 12years (group2). Box plots indicate the median and interquartile range (IQR) and the whiskers represent the maximum and minimum values. **(A)** IgM level on admission; **(B)** IgM level in convalescence; **(C)** IgG level on admission; **(D)** IgG level in convalescence. The results showed lower COVID antibody IgM and IgG level on admission, and IgG level in convalescence [0.13 (0.00, 0.09) vs. 0.12 (0.03, 0.41), *p*<0.05; 0.02 (0.00, 0.14) vs. 1.94 (0.54, 6.40), *p* <0.05; 5.46 (2.41, 9.26) vs. 73.63 (54.63, 86.55), *p*<0.05, respectively], but higher antibody IgM level in convalescence [1.05 (0.51, 2.31) vs. 0.51 (0.20, 1.69), *p*=0.016].

Compared with those aged **≥**12y, the younger group reported lower white blood cell count [5.89 (1.90)×10^9/L vs. 6.45 (2.04)×10^9/L, *p*=0.05], higher lymphocyte count [1.84 (1.32, 2.71)×10^9/L vs. 1.31 (0.94, 1.85)×10^9/L, *p*<0.05), higher normal CRP rate (92.21% vs. 57.72%, *p*<0.05), lower IL-6 levels [5.28 (3.31, 8.13) vs. 9.10 (4.37, 15.14), *p*<0.05], lower levels of COVID-19 antibody IgM and IgG on admission and IgG in convalescence [0.13 (0.00, 0.09) vs. 0.12 (0.03, 0.41), *p*<0.05; 0.02 (0.00, 0.14) vs. 1.94 (0.54, 6.40), *p*<0.05; 5.46 (2.41, 9.26) vs. 73.63 (54.63, 86.55), *p*<0.05, respectively], but higher antibody IgM in convalescence [1.05 (0.51, 2.31) vs. 0.51 (0.20, 1.69), *p*=0.016)] (**Table 2** and **Figure 2**).

Compared with patients aged **≥**12y, the younger group also reported a milder disease status (*p*<0.05), with a higher asymptomatic rate (6.49% vs. 0%), more mild cases (44.16% vs. 32.89%), and fewer moderate and severe and critical cases (49.35% vs. 63.09%; 0% vs. 4.02%, respectively), a lower frequency of pneumonia (53.25% vs. 63.09%, *p*=0.05) and respiratory failure (0% vs. 4.03%), but a longer NAN median, though with no significance [18 (13, 23.5) vs. 16 (12, 21), *p*=0.13]. In the infected patients aged **≥**12y, five patients were admitted to ICU, with a median stay length of nine days [7, 21], two (1.34%) receiving endotracheal intubation; 42 (28.19%) received BRII196/198 therapy; 5 (3.36%) were given convalescent plasma; and two (1.34%) received hormones treatment. However, no such treatments were administered to the children aged<12y (**Table 2**).

### Analysis of Factors Related to NAN

The data were modeled with the R data analysis software. The results showed that the younger group reported a much longer NAN time when compared with patients aged **≥**12y, which was negatively correlated with the IgG level on admission, the days of fever onset after diagnosis, and the nucleic acid CT value (ORF1lab) on admission, but positively with the occurrence of pneumonia, the degree of fever, and the severity of the disease (**Table 3**).

**Table 3:** The effect factors related to NAN time for the patients with SARS-Cov-2 Delta (B.1.167.2) infection.

	Coefficients	Std. Error	P-value
(Intercept)	17.39952	1.96963	<0.0001***
Fever onset after diagnosis	-0.31900	0.12200	0.00957**
IgG level on admission	-0.03822	0.01846	0.03967*
CT value at diagnosis ORF1lab	-0.09493	0.05040	0.05000*
Degrees of Fever			
Low fever	0.83710	0.81097	0.30315
Moderate fever	0.97663	1.08943	0.37103
High fever	2.80145	1.02665	0.00689*
Pneumonia1	3.08877	0.82044	0.00022***
Disease severity			
Mild	1.48801	2.10680	0.48078
Moderate	0.19659	2.17822	0.92817
Severe	2.92080	3.29679	0.37665
Critical	15.03535	3.49264	0.00003***
Group2	-4.71411	1.53909	0.00248**

*indicates that the P value of the variable is less than 0.05.

**indicates that the P value of the variable is less than 0.01.

***indicates that the P value is less than 0.001.

In the exploration of NAN time of patients, because most indicators do not obey normal distribution, this article adopts the method of least squares estimation to evaluate 25 possible influencing variables such as fever duration, type, lymphocyte count, etc. Based on the existing data, this paper uses R data analysis software to model the data, and gradually removes the insignificant influencing factors to obtain the above related factors.

NAN, Nucleic acid negativization.

## Discussion

SARS-CoV-2 B.1.617.2 (Delta variant) first emerged in October 2020 and has spread worldwide (https://www.gisaid.org/hcov19-variants/). The transmission rate of the SARS-COV-2 Delta variant increased by 97% when compared with that of the wild-type strain (Campbell et al., 2021). Patients infected with the Delta variant were twice likely to be hospitalized when compared with those with the Alpha variant (Twohig et al., 2021). In the Guangdong Delta variant surge (May 2021), the viral load in patients was about 1,000 times higher than that of those infected with the original 2019 coronavirus strain (Li et al., 2021). The viral load and transmission speed in the Putian Delta variant surge (Sept. 10^th^, 2021) were similar to those in Guangzhou. But, in the Putian surge, children were the first and prominent spreaders, which was consistent with the findings of other studies. Previous studies showed that school and family were critical transmission places for SARS-COV-2 (Lam-Hine et al., 2021; Madewell et al., 2021), in which the risk of household transmission was expected to be 60% higher for the Delta variant than for the Alpha variant (Mahase, 2021). Previous studies have found that children rarely acted as the main source of secondary transmission in household or school environment (Siebach et al., 2021). However, our research found that in the local surge in Putian, a relatively high proportion of children (up to 34.07%) served as the source of the initial and secondary transmission in schools and the community. This finding suggested that children may be hidden spreaders of COVID-19, which was consistent with the findings of a Norwegian study (Telle et al., 2021).

The Putian Delta variant surge featured younger individuals, milder symptoms and higher vaccination rate. Most of the patients aged ≥ 12y had been vaccinated with two doses of inactivated SARS-COV-2 vaccine while only one-fifth of the patients in the Guangdong Delta surge (May 2021) were vaccinated (Wang et al., 2021). Of note, the current study showed that for patients aged ≥ 12y, the Delta variant-related diseases were much milder in Putian than in the Guangzhou surge, with fewer than one-fifth severe cases (Wang et al., 2021), which was consistent with the results of previous studies (Jara et al., 2021; Lopez Bernal et al., 2021; Sheikh et al., 2021). In addition, the median NAN time was obviously shorter than that of the Guangzhou surge (16 vs. 19 days) (Wang et al., 2021). Further analyses showed that the COVID-19 antibody IgG in the vaccinated population (aged ≥12y) increased much more rapidly and was higher than that of the unvaccinated minor group.

According to the available literature, vaccination contributes to the reduction of the initial virus load upon diagnosis and viral clearance. A recent study has demonstrated that the viral load of fully vaccinated individuals was lower than that of unvaccinated individuals and the load difference started to decline two months after the vaccination and ultimately vanished six months or more after the vaccination (Levine-Tiefenbrun et al., 2021), which signified that viral load was closely related to the duration of vaccination. In our study, most of the vaccinated people had been vaccinated for more than two months and no significant difference in virus load was found between the vaccinated and the unvaccinated population, which was consistent with another study (Chia et al., 2021). The latter reported that compared with unvaccinated individuals, the viral load of fully vaccinated patients decreased faster over time, suggesting the clearance of the virus. Taken together, these findings suggest that vaccination can provide sufficient protection from disease deterioration and temporary protection from COVID infection and accelerate the elimination of virus.

However, the mutation of SARS-COV-2 delta variants may cause immune escape and reduce the sensitivity to neutralizing antibodies, leading to the breakthrough infection of the Delta variants even after the vaccination (Mlcochova et al., 2021). Therefore, the Delta variants may still be potent for quite a long time in the future and unvaccinated children may be critical hidden spreaders due to the absence of effective immunity barrier. In our patient cohort, children aged <12 y were unvaccinated and accounted for more than 1/3 of the total patient population, much more than those in the Guangzhou surge (Wang et al., 2021). In accordance with previous studies, the symptoms of this group were mild as a whole, with milder fever, fewer other symptoms, and no severe cases, when they were compared with those who were over the age of 12 years and vaccinated (Islam et al., 2021; Wang et al., 2021). In this study, the lower level of CRP and IL-6 was observed in the younger children, which may indicate a milder overall inflammatory response and a good prognosis in this population (Liu et al., 2020). The younger children in the current study reported a higher lymphocyte count than their counterparts, which might be due to different physiological status (Yoshida et al., 2021) and different disease conditions between them (Viau & Zouali, 2005; Bieberich et al., 2021)

As for the mild disease status observed in the younger children, a recent study reported that the expression of ACE-2 receptor is lower in the upper and lower airways of children than in the nasal epithelium of healthy adults, resulting in a lower risk of SARS-Cov-2 infection in this population (Yoshida et al., 2021). Furthermore, the milder condition in the younger patient cohort may be closely related to the potent innate immunity of the children. Previous studies (Vono et al., 2021; Winkley et al., 2021; Pierce et al., 2021), indicated that, compared with adults, younger children had a more robust mucosal response to prevent immune escape of the virus and generate an immediate barrier to viral infection. What is more, a recent study documented that the activated interferons in the respiratory mucosa of the children may protect them against SARS-CoV-2 infection (Yoshida et al., 2021), in which children had a significant increase of naive lymphocytes and decrease of NK cells in the systemic response, quite different from adults who had more cytotoxic T-cells (CTL) that were interferon-responsive cells. The presence of fewer interferon-responsive cells in the respiratory tract of the children may also account for a mild inflammatory response in the COVID-19 condition of the children.

However, in contrast to the mild symptoms, the overall median NAN time of children under the age of 12 years was longer than that of those aged 12 years and over. Although studies showed that virus inactivation time was much shorter than the NAN time (Kim et al., 2021), the NAN time was still closely related to the elimination of virus and its infectivity. Further analysis showed that the minor age (<12y) and the COVID-19 IgG level on admission were two of the most important factors for the lengthened NAN time. We hypothesize that as they were unvaccinated, the production of novel antibody was slower in the population aged <12y, resulting in slow virus clearance. These results suggested that if no due attention is paid to children with mild symptoms, they may carry the virus for a longer time and become hidden disseminators of the Delta virus.

Previous studies have showed that a large number of the receptor-binding domain (RBD) specific IgG memory B cells could be induced after a mild infection with COVID-19 virus (Rodda et al., 2021). Children’s B lymphocytes might be activated faster and could produce much more effective antibodies (Vono et al., 2021). The vaccination can produce more memory B lymphocytes in the younger population, which may promote antibody production and speed up virus clearance in the early stage of SARS-CoV-2 infection, so as to reduce their risk as hidden communicators. Therefore, despite the lingering medical disputes, we believe that it is of great significance to promote vaccination in the population aged <12 y in order to combat the novel SARS-CoV-2 variant. It is worth mentioning that, presently, the clinical trials with the mRNA 1273 and bnt162b2 vaccines of Pfizer Company (Ali et al., 2021), and the inactivated vaccines (coronavac) of Sinovac company and Sinopharm Company have been carried out in children and adolescents aged 3-17 y, which has reported a high serum conversion rate of neutralizing antibody (96%~100%) and favorable overall safety (Han et al., 2021; Xia et al., 2021). The Chinese government has recently approved the emergency use of COVID-19 inactivated vaccine in the population aged 3-17 y (Zheng et al., 2021). However, as the immune system of children, especially infants, is in the process of continuous development and improvement and the relevant application data of Covid-19 vaccine is limited at present, more clinical trials should be conducted to clarify the efficacy and risk. Therefore, COVID-19 vaccination policy for children should be implemented on a step by step basis and the effectiveness and adverse events of vaccination should be closely monitored (Zheng et al., 2021).

Some limitations still remain in this study: For one, although the transmission route of all cases was relatively clear and complete, data only came from local patients of Putian, so no multi-center data were accessed; for another, although COVID-19 antibodies IgM and IgG were detected, RBD antibody was not measured and immunofunctional assessments of the effect of T cell-mediated response on the delayed IgG antibody response in children were not conducted. Further studies should be pursued to better evaluate the immune function of children age <12y in order to unravel the underlying mechanisms.

## Data Availability Statement

The original contributions presented in the study are included in the article/**Supplementary Material**. Further inquiries can be directed to the corresponding authors.

## Ethics Statement

The studies involving human participants were reviewed and approved by the Ethics Committee of the Affiliated Hospital of Putian University (No.202152). Written informed consent to participate in this study was provided by the participants’ legal guardian/next of kin.

## Author Contributions

HRL, BX, QYL, and RY contributed to conception and design of the study. HRL, BX, and XC provided methodology. HRL, SL, LH, XL, WZ, QL, and JF organized the database. HRL, XC, and YJ performed the statistical analysis. HRL, HaL, HoL, GC, GZ, SW, XH, HH, and HT contributed to Investigation. BX, HBL, QYL, and RY contributed to project administration. HRL, BX, and HoL edited and revised the manuscript. HRL, HBL, and XC have contributed equally to this work and share first authorship. QYL, RY, and BX are co-corresponding authors. All authors approved the submitted version.

## Funding

The research was funded by the following grants, including Fujian Science and Technology Guidance Project: (2021Y0100), Fujian Natural Science Foundation (2019J01178), Central Government Guiding Local Science and Technology Development (2021L3018), Natural Science Foundation of Fujian Province (2021J01658), High-level hospital foster grants from Fujian Provincial Hospital, Fujian province, China (NO.2019HSJJ11). The Major Health Research Project of Fujian Province (No. 2021ZD01001).

## Conflict of Interest

The authors declare that the research was conducted in the absence of any commercial or financial relationships that could be construed as a potential conflict of interest.

## Publisher’s Note

All claims expressed in this article are solely those of the authors and do not necessarily represent those of their affiliated organizations, or those of the publisher, the editors and the reviewers. Any product that may be evaluated in this article, or claim that may be made by its manufacturer, is not guaranteed or endorsed by the publisher.

## References

[B1] Ali K. Berman G. Zhou H. Deng W. Faughnan V. Coronado-Voges M.. (2021). Evaluation of mRNA-1273 SARS-CoV-2 Vaccine in Adolescents. N Engl. J. Med. 385 (24), 2241–2251. doi: 10.1056/NEJMoa2109522 34379915 PMC8385554

[B2] Baraniuk C. (2021). Covid-19: How Effective are Vaccines Against the Delta Variant? Bmj 374, n1960. doi: 10.1136/bmj.n1960 34373255

[B3] Bieberich F. Vazquez-Lombardi R. Yermanos A. Ehling R. A. Mason D. M. Wagner B.. (2021). A Single-Cell Atlas of Lymphocyte Adaptive Immune Repertoires and Transcriptomes Reveals Age-Related Differences in Convalescent COVID-19 Patients. Front. Immunol. 12. doi: 10.3389/fimmu.2021.701085 PMC8312723 34322127

[B4] Campbell F. Archer B. Laurenson-Schafer H. Jinnai Y. Konings F. Batra N.. (2021). Increased Transmissibility and Global Spread of SARS-CoV-2 Variants of Concern as at June 2021. Euro Surveill 26 (24). doi: 10.2807/1560-7917.Es.2021.26.24.2100509 PMC8212592 34142653

[B5] Cherian S. Potdar V. Jadhav S. Yadav P. Gupta N. Das M.. (2021). Convergent Evolution of SARS-CoV-2 Spike Mutations, L452R, E484Q and P681R, in the Second Wave of COVID-19 in Maharashtra, India. J. bioRxiv. doi: 10.1101/2021.04.22.440932. 2021.2004.2022.440932. PMC8307577 34361977

[B6] Chia P. Y. Xiang Ong S. W. Chiew C. J. Ang L. W. Chavatte J.-M. Mak T.-M.. (2021). Virological and Serological Kinetics of SARS-CoV-2 Delta Variant Vaccine-Breakthrough Infections: A Multi-Center Cohort Study. J. medRxiv. doi: 10.1101/2021.07.28.21261295. 2021.2007.2028.21261295. PMC8608661 34826623

[B7] Coronavirus (COVID-19) Update (2021) FDA Authorizes Pfifizer-BioNTech COVID-19 Vaccine for Emergency Use in Adolescents in Another Important Action in Fight Against Pandemic. Available at: https://www.fda.gov/news-events/press-announcements/coronavirus-covid-19-update-fda-authorizes-pfifizer-biontech-covid-19-vaccine-emergency-use.

[B8] Davies N. G. Abbott S. Barnard R. C. Jarvis C. I. Kucharski A. J. Munday J. D.. (2021). Estimated Transmissibility and Impact of SARS-CoV-2 Lineage B.1.1.7 in England. Science 372 (6538). doi: 10.1126/science.abg3055 PMC8128288 33658326

[B9] Diagnosis and treatment protocol for novel coronavirus pneumonia (Trial Version 8) (2020). Available at: http://www.nhc.gov.cn/yzygj/s7653p/202008/0a7bdf12bd4b46e5bd28ca7f9a7f5e5a.shtml. 10.1097/CM9.0000000000000819 PMC7213636 32358325

[B10] Dyer O. (2021a). Covid-19: Delta Infections Threaten Herd Immunity Vaccine Strategy. Bmj 374, n1933. doi: 10.1136/bmj.n1933 34340962

[B11] Dyer O. (2021b). Covid-19: Unvaccinated Face 11 Times Risk of Death From Delta Variant, CDC Data Show. Bmj 374, n2282. doi: 10.1136/bmj.n2282 34531181

[B12] Fowlkes A. Gaglani M. Groover K. Thiese M. S. Tyner H. Ellingson K. (2021). Effectiveness of COVID-19 Vaccines in Preventing SARS-CoV-2 Infection Among Frontline Workers Before and During B.1.617.2 (Delta) Variant Predominance - Eight U.S. Locations, December 2020-August 2021. MMWR Morb Mortal Wkly Rep. 70 (34), 1167–1169. doi: 10.15585/mmwr.mm7034e4 34437521 PMC8389394

[B13] Grannis S. J. Rowley E. A. Ong T. C. Stenehjem E. Klein N. P. DeSilva M. B.. (2021). Interim Estimates of COVID-19 Vaccine Effectiveness Against COVID-19-Associated Emergency Department or Urgent Care Clinic Encounters and Hospitalizations Among Adults During SARS-CoV-2 B.1.617.2 (Delta) Variant Predominance - Nine States, June-August 2021. MMWR Morb Mortal Wkly Rep. 70 (37), 1291–1293. doi: 10.15585/mmwr.mm7037e2 34529642 PMC8445373

[B14] Griffin S. (2021). Covid-19: Fully Vaccinated People can Carry as Much Delta Virus as Unvaccinated People, Data Indicate. Bmj 374, n2074. doi: 10.1136/bmj.n2074 34413020

[B15] Han B. Song Y. Li C. Yang W. Ma Q. Jiang Z.. (2021). Safety, Tolerability, and Immunogenicity of an Inactivated SARS-CoV-2 Vaccine (CoronaVac) in Healthy Children and Adolescents: A Double-Blind, Randomised, Controlled, Phase 1/2 Clinical Trial. Lancet Infect. Dis. 21 (12), 1645–1653. doi: 10.1016/s1473-3099(21)00319-4 34197764 PMC8238449

[B16] Islam M. A. Kundu S. Alam S. S. Hossan T. Kamal M. A. Hassan R. (2021). Prevalence and Characteristics of Fever in Adult and Paediatric Patients With Coronavirus Disease 2019 (COVID-19): A Systematic Review and Meta-Analysis of 17515 Patients. PloS One 16 (4), e0249788. doi: 10.1371/journal.pone.0249788 33822812 PMC8023501

[B17] Jara A. Undurraga E. A. González C. Paredes F. Fontecilla T. Jara G.. (2021). Effectiveness of an Inactivated SARS-CoV-2 Vaccine in Chile. N Engl. J. Med. 385 (10), 875–884. doi: 10.1056/NEJMoa2107715 34233097 PMC8279092

[B18] Kim M. C. Cui C. Shin K. R. Bae J. Y. Kweon O. J. Lee M. K.. (2021). Duration of Culturable SARS-CoV-2 in Hospitalized Patients With Covid-19. N Engl. J. Med. 384 (7), 671–673. doi: 10.1056/NEJMc2027040 33503337 PMC7934323

[B19] Lam-Hine T. McCurdy S. A. Santora L. Duncan L. Corbett-Detig R. Kapusinszky B.. (2021). Outbreak Associated With SARS-CoV-2 B.1.617.2 (Delta) Variant in an Elementary School - Marin County, California, May-June 2021. MMWR Morb Mortal Wkly Rep. 70 (35), 1214–1219. doi: 10.15585/mmwr.mm7035e2 34473683 PMC8422870

[B20] Levine-Tiefenbrun M. Yelin I. Alapi H. Katz R. Herzel E. Kuint J.. (2021). Viral Loads of Delta-Variant SARS-CoV-2 Breakthrough Infections After Vaccination and Booster With BNT162b2. Nat. Med. 27 (12), 2108–2110. doi: 10.1038/s41591-021-01575-4 34728830

[B21] Li B. Deng A. Li K. Hu Y. Li Z. Xiong Q.. (2021). Viral Infection and Transmission in a Large, Well-Traced Outbreak Caused by the SARS-CoV-2 Delta Variant. Nature Communications. 13 (2), 460. doi: 10.1038/s41467-022-28089-y PMC8786931 35075154

[B22] Liu F. Li L. Xu M. Wu J. Luo D. Zhu Y.. (2020). Prognostic Value of Interleukin-6, C-Reactive Protein, and Procalcitonin in Patients With COVID-19. J. Clin. Virol. 127, 104370. doi: 10.1016/j.jcv.2020.104370 32344321 PMC7194648

[B23] Lopez Bernal J. Andrews N. Gower C. Gallagher E. Simmons R. Thelwall S.. (2021). Effectiveness of Covid-19 Vaccines Against the B.1.617.2 (Delta) Variant. N Engl. J. Med. 385 (7), 585–594. doi: 10.1056/NEJMoa2108891 34289274 PMC8314739

[B24] Madewell Z. J. Yang Y. Longini I. M. Jr. Halloran M. E. Dean N. E. (2021). Factors Associated With Household Transmission of SARS-CoV-2: An Updated Systematic Review and Meta-Analysis. JAMA Netw. Open 4 (8), e2122240. doi: 10.1001/jamanetworkopen.2021.22240 34448865 PMC8397928

[B25] Mahase E. (2021). Delta Variant: What is Happening With Transmission, Hospital Admissions, and Restrictions? Bmj 373, n1513. doi: 10.1136/bmj.n1513 34130949

[B26] Mlcochova P. Kemp S. A. Dhar M. S. Papa G. Meng B. Ferreira I.. (2021). SARS-CoV-2 B.1.617.2 Delta Variant Replication and Immune Evasion. Nat. doi: 10.1038/s41586-021-03944-y PMC8566220 34488225

[B27] Pierce C. A. Sy S. Galen B. Goldstein D. Y. Orner E. Keller M. J.. (2021). Natural Mucosal Barriers and COVID-19 in Children. JCI Insight 6 (9). doi: 10.1172/jci.insight.148694 PMC8262299 33822777

[B28] Press Release (2021) JCVI Issues Advice on COVID-19 Vaccination of Children and Young People. Available at: https://www.gov.uk/government/news/jcvi-issues-advice-on-covid-19-vaccination-of-children-and-young-people.

[B29] Pritchard E. Matthews P. C. Stoesser N. Eyre D. W. Gethings O. Vihta K. D.. (2021). Impact of Vaccination on New SARS-CoV-2 Infections in the United Kingdom. Nat. Med. 27 (8), 1370–1378. doi: 10.1038/s41591-021-01410-w 34108716 PMC8363500

[B30] Pung R. Mak T. M. Kucharski A. J. Lee V. J. (2021). Serial Intervals in SARS-CoV-2 B.1.617.2 Variant Cases. Lancet 398 (10303), 837–838. doi: 10.1016/s0140-6736(21)01697-4 34388398 PMC8354568

[B31] Rodda L. B. Netland J. Shehata L. Pruner K. B. Morawski P. A. Thouvenel C. D.. (2021). Functional SARS-CoV-2-Specific Immune Memory Persists After Mild COVID-19. Cell 184 (1), 169–183.e117. doi: 10.1016/j.cell.2020.11.029 33296701 PMC7682481

[B32] Schroeder A. R. Wilson K. M. Ralston S. L. (2020). COVID-19 and Kawasaki Disease: Finding the Signal in the Noise. Hosp Pediatr. 10 (10), e1–e3. doi: 10.1542/hpeds.2020-000356 32404331

[B33] Sheikh A. McMenamin J. Taylor B. Robertson C. (2021). SARS-CoV-2 Delta VOC in Scotland: Demographics, Risk of Hospital Admission, and Vaccine Effectiveness. Lancet 397 (10293), 2461–2462. doi: 10.1016/s0140-6736(21)01358-1 34139198 PMC8201647

[B34] Shiri T. Evans M. Talarico C. A. Morgan A. R. Mussad M. Buck P. O.. (2021). Vaccinating Adolescents and Children Significantly Reduces COVID-19 Morbidity and Mortality Across All Ages: A Population-Based Modeling Study Using the UK as an Example. Vaccines (Basel) 9 (10). doi: 10.3390/vaccines9101180 PMC8537561 34696288

[B35] Siebach M. K. Piedimonte G. Ley S. H. (2021). COVID-19 in Childhood: Transmission, Clinical Presentation, Complications and Risk Factors. Pediatr. Pulmonol 56 (6), 1342–1356. doi: 10.1002/ppul.25344 33721405 PMC8137603

[B36] Telle K. Jørgensen S. B. Hart R. Greve-Isdahl M. Kacelnik O. (2021). Secondary Attack Rates of COVID-19 in Norwegian Families: A Nation-Wide Register-Based Study. Eur. J. Epidemiol. 36 (7), 741–748. doi: 10.1007/s10654-021-00760-6 34036466 PMC8147908

[B37] Twohig K. A. Nyberg T. Zaidi A. Thelwall S. Sinnathamby M. A. Aliabadi S.. (2021). Hospital Admission and Emergency Care Attendance Risk for SARS-CoV-2 Delta (B.1.617.2) Compared With Alpha (B.1.1.7) Variants of Concern: A Cohort Study. Lancet Infect. Dis. doi: 10.1016/s1473-3099(21)00475-8 PMC8397301 34461056

[B38] Viau M. Zouali M. (2005). B-Lymphocytes, Innate Immunity, and Autoimmunity. Clin. Immunol. 114 (1), 17–26. doi: 10.1016/j.clim.2004.08.019 15596405

[B39] Vono M. Huttner A. Lemeille S. Martinez-Murillo P. Meyer B. Baggio S.. (2021). Robust Innate Responses to SARS-CoV-2 in Children Resolve Faster Than in Adults Without Compromising Adaptive Immunity. Cell Rep. 37 (1), 109773. doi: 10.1016/j.celrep.2021.109773 34587479 PMC8440231

[B40] Wang Y. Chen R. Hu F. Lan Y. Yang Z. Zhan C.. (2021). Transmission, Viral Kinetics and Clinical Characteristics of the Emergent SARS-CoV-2 Delta VOC in Guangzhou, China. EClinicalMedicine 40, 101129. doi: 10.1016/j.eclinm.2021.101129 34541481 PMC8435265

[B41] Winkley K. Banerjee D. Bradley T. Koseva B. Cheung W. A. Selvarangan R.. (2021). Immune Cell Residency in the Nasal Mucosa may Partially Explain Respiratory Disease Severity Across the Age Range. Sci. Rep. 11 (1), 15927. doi: 10.1038/s41598-021-95532-3 34354210 PMC8342554

[B42] Xia S. Zhang Y. Wang Y. Wang H. Yang Y. Gao G. F.. (2021). Safety and Immunogenicity of an Inactivated COVID-19 Vaccine, BBIBP-CorV, in People Younger Than 18 Years: A Randomised, Double-Blind, Controlled, Phase 1/2 Trial. Lancet Infect. Dis. doi: 10.1016/s1473-3099(21)00462-x PMC8443232 34536349

[B43] Yonker L. M. Neilan A. M. Bartsch Y. Patel A. B. Regan J. Arya P.. (2020). Pediatric Severe Acute Respiratory Syndrome Coronavirus 2 (SARS-CoV-2): Clinical Presentation, Infectivity, and Immune Responses. J. Pediatr. 227, 45–52.e45. doi: 10.1016/j.jpeds.2020.08.037 32827525 PMC7438214

[B44] Yoshida M. Worlock K. B. Huang N. Lindeboom R. G. H. Butler C. R. Kumasaka N.. (2021). Local and Systemic Responses to SARS-CoV-2 Infection in Children and Adults. Nature. doi: 10.1038/s41586-021-04345-x PMC8828466 34937051

[B45] Zheng Y. J. Wang X. C. Feng L. Z. Xie Z. D. Jiang Y. Lu G.. (2021). Expert Consensus on COVID-19 Vaccination in Children. World J. Pediatr. 17 (5), 449–457. doi: 10.1007/s12519-021-00465-6 34618327 PMC8494629

