# Supplementary material for: Unvaccinated Children Are an Important Link in the Transmission of SARS-CoV-2 Delta Variant (B1.617.2): Comparative Clinical Evidence From a Recent Community Surge
Source: Front Cell Infect Microbiol. 2022 Mar 8;12:814782. doi: 10.3389/fcimb.2022.814782 (PMC8957884; doi:10.3389/fcimb.2022.814782)
Supplement: Supplementary file 1 [file Table_1_v1.doc]

| Number | Test Item | Apparatus | Kit |
| --- | --- | --- | --- |
| 1 | Blood test | SYSME, XE-5000 or XE-2100, Japan | Corollary Reagent |
| 2 | Biochemistry analysis | Mindary, BS-800M, China | Corollary Reagent |
| 3 | CRP | Boditech Med Inc, A5000, China | Corollary Reagent |
| 4 | SARS-CoV-2 IgG/IgM antibody titer | Bioscience, Axceed260, China | Corollary Reagent |
| 5 | Humoral immunity test | Aristo, China | Corollary Reagent |
| 6 | IL-6 | GeteinBiotech, GP1600, China | Corollary Reagent |
| 7 | Urine test | Sysmex, UF-1000i, Japan | Corollary Reagent |
| 8 | Blood coagulation function | STAGO, COMPACT, France | Corollary Reagent |
| 9 | Lymphocyte and subsets count | BD, BD FACSCalibur, American | BD Multitest IMK Kit |
| 10 | Cytokines | BD, BD FACSCalibur, American | Ralsecare, China |
| 11 | SARS-Cov-2 RT-PCR | Nucleic Acid Extraction System：Bioer(NPA-32P)  PCR Amplifier：King Dian(MC-1000) | Extraction kit： Bioer  Nucleic acid amplification Kit: Shanghai ZJ |
| 12 | Microbe testing | BioMérieux Recruitment, China | Corollary Reagent |

**Test Items and Apparatus (Kit)**
